# Paediatric Splenic Abscess Due to Salmonella

**DOI:** 10.1590/0037-8682-0093-2024

**Published:** 2024-07-29

**Authors:** Kemal Buğra Memiş, Zeynep Betul Deve, Sonay Aydın

**Affiliations:** 1 Erzincan University, School of Medicine, Department of Radiology, Erzincan, Turkey.

A 12-year-old boy presented to the emergency department with upper left quadrant abdominal pain, fever, and vomiting for three days. He had a fever of 38.5 °C. Test results revealed an elevated white blood cell count and C-reactive protein level.

**FIGURE 1:**
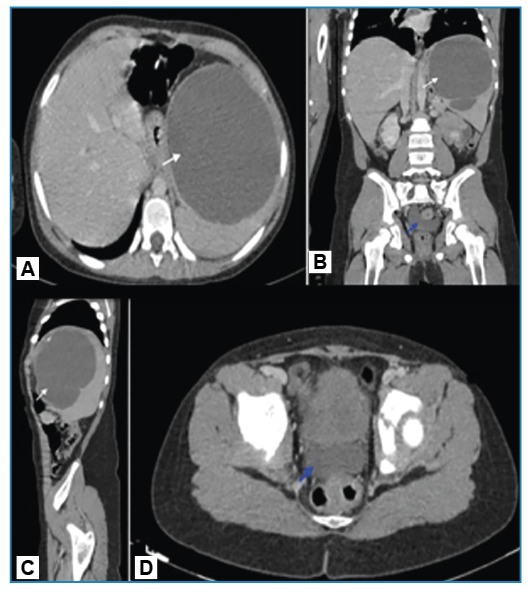
Axial- **(A)**, coronal- **(B)** and sagittal**-**plane **(C)** abdominal CT showing a thick-walled hypodense cystic lesion approximately 11 cm in diameter in the spleen, with septations and wall calcification in the superior aspect **(white arrows)**; axial- **(D)** and coronal-plane **(B)** abdominal CT showing free fluid of approximately 2 cm depth in the pelvic area **(blue arrow)**.

**FIGURE 2:**
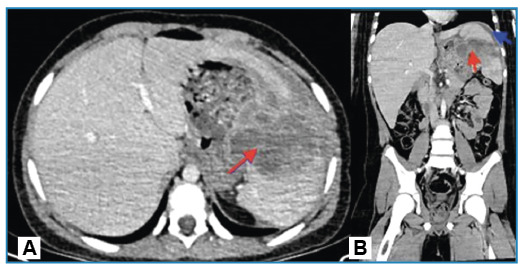
Axial- **(A)** and coronal-plane **(B)** CT images of a hematoma occurring in the perisplenic region one month after the drainage. 7x5-cm septated and wall-enhancing collection area, extending into the splenogastric recess **(red arrows)**; free fluid measuring 15 mm in depth in the perisplenic space **(blue arrow)**.

Computed tomography (CT) revealed a thick-walled cystic lesion measuring approximately 11 cm across the spleen. The patient experienced septation and wall calcification on the upper side. A 2-cm depth of pelvic free fluid was also found (Figure 1). Percutaneous drainage and an 8-day antibiotic regimen were administered, and *Salmonella* type B was cultured from the abscess. The symptoms improved, leading to discharge. Follow-up CT revealed a 7 × 5-cm septate collection area extending into the splenogastric recess, compressing the stomach’s greater curvature and causing inflammatory wall thickening with perisplenic free fluid, suggestive of a hematoma (Figure 2). Following discharge, the hematoma, which was monitored using ultrasonography in our radiology department, was completely absorbed.

Splenic abscesses are rare in children and are associated with high mortality rates. The reported incidence in autopsy series ranges from 0.14%-0.7%^1^. Symptoms such as fever and abdominal pain are nonspecific. Increased imaging allows for early diagnosis, which is crucial. Aerobic microorganisms, including staphylococci, streptococci, salmonella, and *Escherichia coli*, are common^2^. Percutaneous drainage, preferred over surgery, yields 51-72% success rates under specific conditions, replacing traditional antibiotic therapy and splenectomy^3^ ^,^ ^4^. This case highlights the importance of considering splenic abscesses in the differential diagnosis of children presenting with nonspecific symptoms such as fever and abdominal pain.
